# Making memories

**DOI:** 10.7554/eLife.102837

**Published:** 2024-10-02

**Authors:** Francesco Longo

**Affiliations:** 1 Department of Pharmacology, University of Gothenburg Gothenburg Sweden; 2 Institute of Neuroscience and Physiology, University of Gothenburg Gothenburg Sweden; 3 Sahlgreska Academy, University of Gothenburg Gothenburg Sweden

**Keywords:** dopamine, LTP, memory, cell signalling, protein synthesis

## Abstract

The neurotransmitter dopamine helps form long-term memories by increasing the production of proteins through a unique signaling pathway.

**Related research article** Fuchsberger T, Stockwell I, Woods M, Brzosko Z, Greger IH, Paulsen O. 2024. Dopamine increases protein synthesis in hippocampal neurons enabling dopamine-dependent LTP. *eLife* **13**:RP100822. doi: 10.7554/eLife.100822.

Dopamine is often referred to as the ‘feel-good’ neurotransmitter. Whether it is the satisfaction of a delicious meal, the thrill of success, or the drive to accomplish a goal – dopamine is central to our ability to experience pleasure, stay motivated and feel rewarded (Salamone et al., 2003; Schultz, 1997). However, it is also vital to how we learn and form lasting memories (Shohamy and Adcock, 2010; Takeuchi et al., 2016). So, how exactly does this neurotransmitter help lock memories in place?

When something new is learned, the synapses involved in storing the information become stronger through a process known as long-term potentiation (LTP), which relies on increased protein production. This results in the memory being more stable and easier to retrieve (Santini et al., 2014). Previous research, including work conducted by the research group led by Ole Paulsen, has shown that dopamine is critical for LTP, and that it can also regulate protein synthesis in neurons in the hippocampus, a brain region important for memory (Smith et al., 2005; Fuchsberger et al., 2022). Now, in eLife, Ole Paulsen and colleagues – including Tanja Fuchsberger as first author – report having identified a unique signaling pathway by which dopamine triggers the protein synthesis required to form long-term memories (Fuchsberger et al., 2024).

First, the team (who are based at the University of Cambridge and the MRC Laboratory of Molecular Biology) used a labelling technique to detect newly synthesized proteins in slices of the mouse hippocampus. This revealed that when dopamine was present, protein production significantly increased in CA1 neurons important for memory formation.

Further experiments showed that dopamine could transform long-term depression – a process that usually weakens synapses – into LTP, effectively converting weak synaptic connections into stronger ones. However, when protein synthesis was blocked, this ability disappeared. When dopamine was applied during neuronal stimulation, the synapses not only maintained their strength but grew even stronger, suggesting neuronal activation also contributes to the dopamine-induced increase in protein synthesis.

Fuchsberger et al. found that this strengthening of synaptic connections was mediated by two dopamine receptors (D1 and D5) that trigger a cascade of signals inside neurons. This includes activating enzymes known as adenylate cyclases, which simulate two other signaling molecules (cAMP and PKA), ultimately leading to a boost in protein production (Figure 1; Sassone-Corsi, 2012).

**Figure 1.**
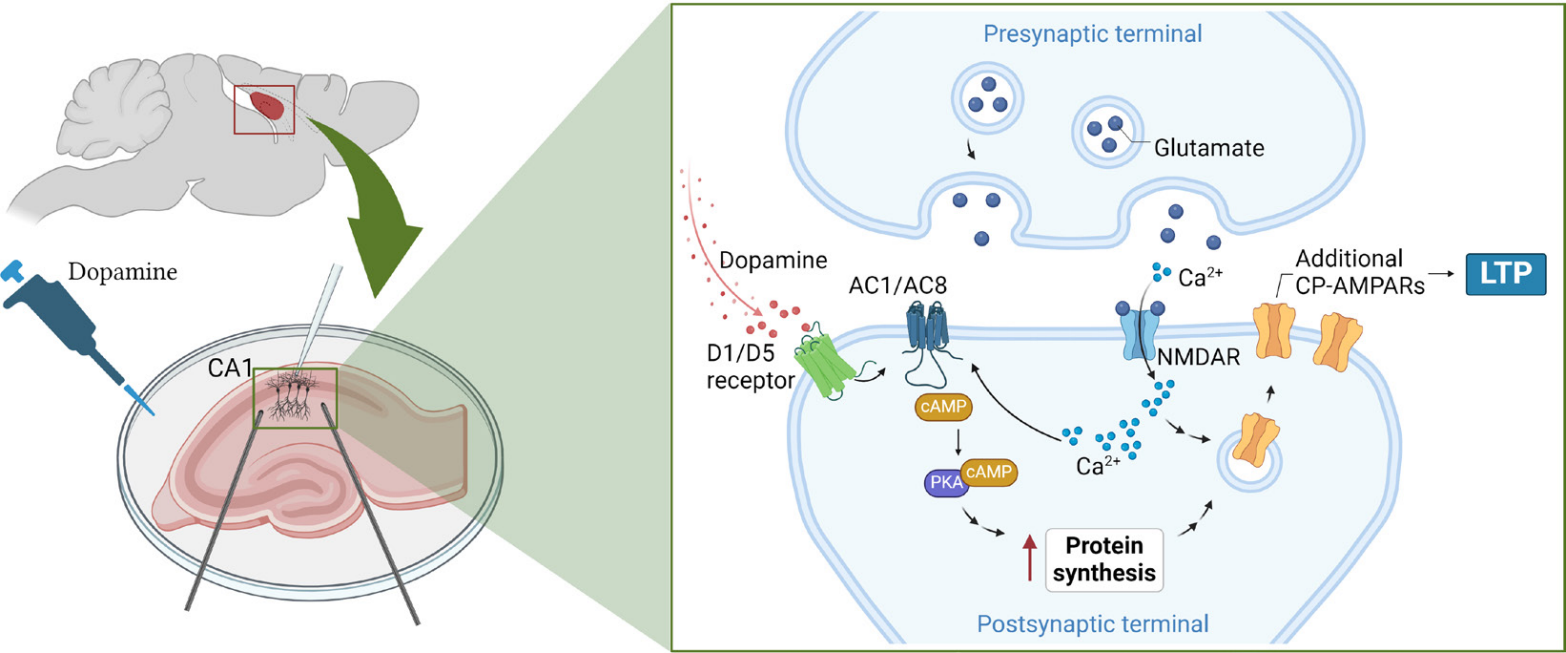
How dopamine increases synaptic strength in the hippocampus. (Left) To test the effects of dopamine on long-term potentiation (LTP), Fuchsberger et al. injected the neurotransmitter into slices of the hippocampus (pink) and recorded the electrical activity of CA1 neurons (white electrode). (Right inset) They found that dopamine triggers the D1/D5 receptor (green) to activate a signaling pathway which includes the enzymes AC1/AC8 (dark blue), cAMP (brown) and PKA (purple). This leads to the production of more proteins that support the strengthening of the synapse and long-term potentiation. At the same time, neurotransmitters released from the pre-synaptic terminal, such as glutamate (dark blue circles), stimulate proteins on the post-synaptic terminal, such as NMDA receptors (light blue), to transmit calcium ions (Ca^2+^; light blue small circles) into the neuron. The calcium ions also activate AC1/AC8, ensuring that dopamine-induced protein synthesis is paired with neural activation. This increases the production of GluA1, a type of subunit that can assemble to form calcium-permeable AMPA receptors (yellow), which Fuchsberger et al. show to be essential for dopamine-induced LTP. AC1/AC8: adenylate cyclase 1/8; cAMP: cyclic adenosine monophosphate; PKA: protein kinase A. Figure 1 was created with BioRender.com.

Genetically removing two types of adenylate cyclase enzymes – called AC1 and AC8 – from CA1 neurons led to dopamine-induced LTP being completely absent. These enzymes are also activated by calcium ions that flood into the synapse during neuronal activation, suggesting that they couple neuronal activity and dopamine signaling in order to induce protein synthesis (Figure 1).

Fuchsberger et al. found that dopamine increased the production of the protein GluA1, a subunit found in AMPA receptors which support LTP. However, the neurotransmitter had no effect on GluA2, another type of subunit found in AMPA receptors.

AMPA receptors formed of only GluA1 subunits have captivated researchers due to their unique structures and signaling properties (Zhang et al., 2023), and the fact that, unlike AMPA receptors featuring other subunits, they allow calcium ions to pass through them (Park et al., 2019). Fuchsberger et al. discovered that when AMPA receptors containing just GluA1 were blocked, dopamine could no longer strengthen synaptic connections, suggesting that these calcium-permeable receptors are needed for dopamine-induced LTP (Figure 1).

These findings offer a fresh perspective on how dopamine influences, not only our motivation and mood, but also our ability to learn and remember. They show which signaling pathways and molecules allow dopamine to induce the protein synthesis required for long-term memory. This opens up exciting possibilities for targeting dopamine pathways in therapies designed to improve memory and learning, particularly for conditions like Alzheimer’s disease where memory formation is impaired. Modulating the effect of dopamine could also help reinforce memory in individuals experiencing cognitive decline.

One of the key challenges ahead is to fully understand the molecular mechanisms and cell-type specificity of dopamine’s influence on protein synthesis in neurons. Timing is also likely to play a crucial role, and understanding when dopamine is released in relation to neuronal activity may reveal critical periods for optimizing learning and memory processing. Future research exploring these temporal dynamics could shed light on the best approaches for harnessing dopamine’s full potential in enhancing memory and cognitive function.
